# Supplementary material for: Humoral immunogenicity of a Coronavirus Disease 2019 (COVID-19) DNA vaccine in rhesus macaques (Macaca mulatta) delivered using needle-free jet injection
Source: PLoS One. 2023 May 31;18(5):e0275082. doi: 10.1371/journal.pone.0275082 (PMC10231776; doi:10.1371/journal.pone.0275082)
Supplement: S1 Data — (DOCX) [file pone.0275082.s001.docx]

**Supplemental data**

**Figure 1 data tables:**

| Day 0 |  |  |  |  |  |  |
| --- | --- | --- | --- | --- | --- | --- |
| Animal ID | PRNT50 | PsVNA50 | Full Spike | S1 | RBD | N |
| 1 | 14.1 | 14.1 | 1 | 1 | 1 | 1 |
| 2 | 14.1 | 14.1 | 1 | 1 | 1 | 1 |
| 3 | 14.1 | 14.1 | 1 | 1 | 1 | 1 |
| 4 | 14.1 | 14.1 | 1 | 1 | 1 | 1 |
| 5 | 14.1 | 14.1 | 1 | 1 | 1 | 1 |
| 6 | 14.1 | 14.1 | 1 | 1 | 1 | 1 |
| 7 | 14.1 | 14.1 | 1 | 1 | 1 | 1 |
| 8 | 14.1 | 14.1 | 1 | 1 | 1 | 1 |
| 9 | 14.1 | 14.1 | 1 | 1 | 1 | 1 |
| 10 | 14.1 | 14.1 | 1 | 1 | 1 | 1 |
| 11 | 14.1 | 14.1 | 1 | 1 | 1 | 1 |
| 12 | 14.1 | 14.1 | 1 | 1 | 1 | 1 |
|  |  |  |  |  |  |  |
| Day 21 |  |  |  |  |  |  |
|  | PRNT50 | PsVNA50 | Full Spike | S1 | RBD | N |
| 703080 | 14.1 | 14.1 | 1 | 1.1 | 1 | 0.9 |
| 712289 | 14.1 | 14.1 | 0.6 | 0.8 | 1 | 0.8 |
| T1361205 | 14.1 | 39 | 8.4 | 20.6 | 1.4 | 1.1 |
| T126255 | 40 | 96 | 8.9 | 17.1 | 43.1 | 0.9 |
| 804039 | 14.1 | 14.1 | 0.4 | 0.4 | 9.5 | 1.3 |
| 806091 | 14.1 | 14.1 | 1.4 | 1.1 | 1.3 | 1.1 |
| 32466 | 14.1 | 14.1 | 1.2 | 2.2 | 2.9 | 1 |
| T1461711 | 14.1 | 14.1 | 1.2 | 0.9 | 1 | 1 |
| 807001 | 14.1 | 14.1 | 0.9 | 0.9 | 0.9 | 1.1 |
| T126601 | 14.1 | 14.1 | 0.9 | 0.9 | 0.9 | 1.2 |
| T1461713 | 14.1 | 14.1 | 1.1 | 1.2 | 1.4 | 1.1 |
| 7092561 | 14.1 | 14.1 | 0.8 | 0.9 | 0.9 | 1.2 |
|  |  |  |  |  |  |  |
| Day 35 |  |  |  |  |  |  |
|  | PRNT50 | PsVNA50 | Full Spike | S1 | RBD | N |
| 703080 | 14.1 | 68 | 5.4 | 18.5 | 9.3 | 0.9 |
| 712289 | 80 | 306 | 28 | 118.3 | 205.2 | 0.8 |
| T1361205 | 14.1 | 32 | 20.4 | 66.4 | 5.7 | 1.1 |
| T126255 | 20 | 41 | 16.9 | 28.7 | 43.6 | 1 |
| 804039 | 40 | 62 | 9.8 | 2 | 354.9 | 1.3 |
| 806091 | 14.1 | 14.1 | 2.8 | 6.2 | 10.3 | 1.1 |
| 32466 | 20 | 41 | 5.2 | 23.3 | 14.6 | 1 |
| T1461711 | 20 | 14.1 | 2.4 | 1.2 | 2.3 | 0.9 |
| 807001 | 14.1 | 14.1 | 1.3 | 0.8 | 1.1 | 1.2 |
| T126601 | 14.1 | 14.1 | 0.9 | 0.9 | 0.9 | 1.4 |
| T1461713 | 14.1 | 14.1 | 2.3 | 1.3 | 1.4 | 1 |
| 7092561 | 14.1 | 14.1 | 2.4 | 1 | 1.1 | 1.4 |
|  |  |  |  |  |  |  |
| Day 63 |  |  |  |  |  |  |
|  | PRNT50 | PsVNA50 | Full Spike | S1 | RBD | N |
| 703080 | 40 | 104 | 17.7 | 72.4 | 31.6 | 0.8 |
| 712289 | 320 | 1751 | 66.2 | 452.2 | 494.3 | 1.5 |
| T1361205 | 80 | 408 | 52 | 183.3 | 22.6 | 1.1 |
| T126255 | 80 | 107 | 87.2 | 42.7 | 90.1 | 1 |
| 804039 | 40 | 335 | 22.2 | 17.4 | 430.9 | 1.2 |
| 806091 | 40 | 443 | 17.8 | 187.2 | 190.9 | 1.2 |
| 32466 | 40 | 185 | 12.4 | 76.5 | 38.7 | 1 |
| T1461711 | 14.1 | 14.1 | 25 | 29.1 | 27.6 | 0.9 |
| 807001 | 14.1 | 92 | 70.2 | 128.1 | 10.2 | 0.2 |
| T126601 | 14.1 | 14.1 | 4.7 | 5.4 | 3.1 | 1.4 |
| T1461713 | 14.1 | 14.1 | 46.3 | 4.7 | 3.7 | 0.8 |
| 7092561 | 14.1 | 14.1 | 12.8 | 6.6 | 2.5 | 1.1 |

**Figure 2 data tables:**

| PRNT50 |  |  |  |  |  |  |  |  |  |  |  |  |
| --- | --- | --- | --- | --- | --- | --- | --- | --- | --- | --- | --- | --- |
|  | Animal ID | |  |  |  |  |  |  |  |  |  |  |
| Day | 1 | 2 | 3 | 4 | 5 | 6 | 7 | 8 | 9 | 10 | 11 | 12 |
| 0 | 14.1 | 14.1 | 14.1 | 14.1 | 14.1 | 14.1 | 14.1 | 14.1 | 14.1 | 14.1 | 14.1 | 14.1 |
| 21 | 14.1 | 14.1 | 14.1 | 40 | 14.1 | 14.1 | 14.1 | 14.1 | 14.1 | 14.1 | 14.1 | 14.1 |
| 35 | 14.1 | 80 | 14.1 | 20 | 40 | 14.1 | 20 | 20 | 14.1 | 14.1 | 14.1 | 14.1 |
| 63 | 40 | 320 | 80 | 80 | 40 | 40 | 40 | 14.1 | 14.1 | 14.1 | 14.1 | 14.1 |
| 168 | 14.1 | 40 | 20 | 14.1 | 20 | 14.1 | 20 | 14.1 | 14.1 | 14.1 | 14.1 | 14.1 |
|  |  |  |  |  |  |  |  |  |  |  |  |  |
| PsVNA50 |  |  |  |  |  |  |  |  |  |  |  |  |
|  | Animal ID | |  |  |  |  |  |  |  |  |  |  |
| Day | 1 | 2 | 3 | 4 | 5 | 6 | 7 | 8 | 9 | 10 | 11 | 12 |
| 0 | 14.1 | 14.1 | 14.1 | 14.1 | 14.1 | 14.1 | 14.1 | 14.1 | 14.1 | 14.1 | 14.1 | 14.1 |
| 21 | 14.1 | 14.1 | 39 | 96 | 14.1 | 14.1 | 14.1 | 14.1 | 14.1 | 14.1 | 14.1 | 14.1 |
| 35 | 68 | 306 | 32 | 41 | 62 | 14.1 | 41 | 14.1 | 14.1 | 14.1 | 14.1 | 14.1 |
| 42 | 28 | 750 | 91 | 28 | 50 | 14.1 | 95 | 14.1 | 14.1 | 14.1 | 14.1 | 14.1 |
| 63 | 104 | 1751 | 408 | 107 | 335 | 443 | 185 | 14.1 | 92 | 14.1 | 14.1 | 14.1 |
| 168 | 87 | 102 | 143 | 14.1 | 98 | 88 | 67 | 14.1 | 14.1 | 49 | 14.1 | 37 |

Figure 3 data tables:

| PRNT50 |  |  |  |  |
| --- | --- | --- | --- | --- |
| Animal ID | WA1 PRNT50 | Gamma PRNT50 | Delta PRNT50 |  |
| 1 | 40 | 20 | 20 |  |
| 2 | 320 | 40 | 80 |  |
| 3 | 80 | 40 | 40 |  |
| 4 | 80 | 20 | 20 |  |
| 5 | 40 | 20 | 20 |  |
| 6 | 40 | 20 | 80 |  |
| 7 | 40 | 40 | 80 |  |
| 8 | 14.1 | 20 | 20 |  |
| 9 | 14.1 | 14.1 | 14.1 |  |
| 10 | 14.1 | 14.1 | 14.1 |  |
| 11 | 14.1 | 14.1 | 40 |  |
| 12 | 14.1 | 40 | 20 |  |
|  |  |  |  |  |
| PsVNA50 |  |  |  |  |
|  | WA1 | Beta | Delta | OMI |
| 1 | 104 | 94 | 161 | 14.1 |
| 2 | 1751 | 256 | 1942 | 14.1 |
| 3 | 408 | 236 | 485 | 21 |
| 4 | 107 | 90 | 536 | 33 |
| 5 | 335 | 83 | 259 | 37 |
| 6 | 443 | 242 | 1267 | 151 |
| 7 | 185 | 186 | 2190 | 14.1 |
| 8 | 14.1 | 81 | 14.1 | 14.1 |
| 9 | 92 | 79 | 324 | 14.1 |
| 10 | 14.1 | 20 | 14.1 | 36 |
| 11 | 14.1 | 14.1 | 34 | 14.1 |
| 12 | 14.1 | 110 | 49 | 14.1 |
